# Integration of Light and Temperature in the Regulation of Circadian Gene Expression in *Drosophila*

**DOI:** 10.1371/journal.pgen.0030054

**Published:** 2007-04-06

**Authors:** Catharine E Boothroyd, Herman Wijnen, Felix Naef, Lino Saez, Michael W Young

**Affiliations:** 1 Laboratory of Genetics, The Rockefeller University, New York, New York, United States of America; 2 Department of Biology, University of Virginia, Charlottesville, Virginia, United States of America; 3 Laboratory of Mathematical Physics, The Rockefeller University, New York, New York, United States of America; 4 School of Life Sciences, Ecole Polytechnique Fédérale de Lausanne, Lausanne, Switzerland; North Carolina State University, United States of America

## Abstract

Circadian clocks are aligned to the environment via synchronizing signals, or *Zeitgebers,* such as daily light and temperature cycles, food availability, and social behavior. In this study, we found that genome-wide expression profiles from temperature-entrained flies show a dramatic difference in the presence or absence of a thermocycle. Whereas transcript levels appear to be modified broadly by changes in temperature, there is a specific set of temperature-entrained circadian mRNA profiles that continue to oscillate in constant conditions. There are marked differences in the biological functions represented by temperature-driven or circadian regulation. The set of temperature-entrained circadian transcripts overlaps significantly with a previously defined set of transcripts oscillating in response to a photocycle. In follow-up studies, all thermocycle-entrained circadian transcript rhythms also responded to light/dark entrainment, whereas some photocycle-entrained rhythms did not respond to temperature entrainment. Transcripts encoding the clock components Period, Timeless, Clock, Vrille, PAR-domain protein 1, and Cryptochrome were all confirmed to be rhythmic after entrainment to a daily thermocycle, although the presence of a thermocycle resulted in an unexpected phase difference between *period* and *timeless* expression rhythms at the transcript but not the protein level. Generally, transcripts that exhibit circadian rhythms both in response to thermocycles and photocycles maintained the same mutual phase relationships after entrainment by temperature or light. Comparison of the collective temperature- and light-entrained circadian phases of these transcripts indicates that natural environmental light and temperature cycles cooperatively entrain the circadian clock. This interpretation is further supported by comparative analysis of the circadian phases observed for temperature-entrained and light-entrained circadian locomotor behavior. Taken together, these findings suggest that information from both light and temperature is integrated by the transcriptional clock mechanism in the adult fly head.

## Author Summary

A key adaptation to life on Earth is provided by internal daily time-keeping mechanisms that allow anticipation of the alternations between night and day. To act as reliable time-keeping mechanisms, circadian clocks have to be able to synchronize to environmental time cues, maintain ∼24-h rhythms under constant conditions, run at approximately the same pace over a range of environmental temperatures, and efficiently communicate time-of-day information to other biological systems. Clock-controlled oscillations in gene expression play an essential role in producing overt circadian rhythms. For most organisms, light/dark cycles appear to constitute the most powerful entrainment cue, but daily temperature cycles have also been demonstrated to efficiently synchronize circadian rhythms. This study uses the fruit fly Drosophila melanogaster as a model to compare the clock-dependent and clock-independent daily gene expression rhythms produced in response to light/dark cycles versus temperature cycles. A broad temperature-driven expression program was found in the heads of both wild-type and arrhythmic mutant flies, but wild-type flies also exhibited a more specific temperature-entrained circadian expression response that resembled the circadian response following light entrainment. The phase relationship between the temperature- and light-entrained circadian rhythms suggests that in nature light and temperature act cooperatively to synchronize the circadian clock.

## Introduction

Organisms on Earth have evolved an internal timekeeping system, or circadian clock (*circa* = about, *diem* = day), that allows them to both respond to and predict changes in the 24-h environmental day. Much has been learned about the genes involved in this precise, 24-h molecular timekeeping mechanism in the fruit fly Drosophila melanogaster (for a recent review see [1]). The fly clock is composed of intracellular feedback loops: The proteins Clock (CLK) and Cycle (CYC) activate transcription of *period (per), timeless (tim), vrille (vri),* and *PAR-domain protein 1 (Pdp1).* Subsequently, proteins encoded by the latter four genes either suppress or activate CLK and CYC [2–8]. Feedback in these regulatory loops is thought to oscillate due to timed changes in the stabilities and subcellular localizations of component proteins, especially Period (PER) and Timeless (TIM) [9,10].

The fly molecular clock is aligned to the environment through *Zeitgebers* (“time givers”), the most notable being the daily light/dark cycle. This is mediated by the light-dependent degradation of the TIM protein [11,12]. Cryptochrome (CRY), a blue light photoreceptor in the family of flavoproteins, has been shown to associate with TIM during the light phase of the circadian day, resulting in ubiquitination and degradation of TIM by the proteasome and ultimately relieving inhibition of CLK-mediated transcription [13–15]. In addition, a second pathway of light entrainment in the pacemaker neurons is defined by signals from visual organs that may impact TIM in a CRY-independent manner [13,16].

Light is the best understood *Zeitgeber,* but other factors, such as daily changes in temperature [17–20] and social behavior [21], can act as inputs to the fly circadian clock. Although the fly clock is temperature-compensated over a wide range of constant physiological temperatures, it has been known for several decades that eclosion in Drosophila pseudoobscura can entrain cycling temperature changes [20]. Further, it was shown in this species that temperature step-ups, step-downs, and pulses result in accompanying phase shifts in behavior [22]. Locomotor activity behavior in D. melanogaster can be entrained to temperature cycles of as little as 3 °C [18]. The locomotor activity rhythms of arrhythmic clock mutants *(per^0^, tim^01^, Clk^Jrk^, cyc^0^)* can be driven by temperature [19]. However, these “clock-less” mutants do not truly entrain as there is no anticipation of the temperature transitions, and rhythmicity does not persist when they are released into constant conditions. Interestingly, the locomotor activity of wild-type flies can be entrained to temperature cycles during constant light, a condition that would normally result in arrhythmicity [17,19,23]. There is anticipation of temperature transitions, but as with the arrhythmic clock mutants, locomotor activity behavior becomes arrhythmic when the temperature cycle is removed.

Molecularly it has been shown that short, high temperature heat pulses result in rapid downregulation of both PER and TIM proteins [24]. This results in a phase delay if the heat pulse is administered in the early night. However, a heat pulse given in the late night does not result in a phase advance, as is the case with a light pulse given at this time. This is thought to be due to a rapid increase in PER and TIM production after the initial downregulation, ultimately resulting in a constant period [24]. It is not clear if the molecular responses triggered by abrupt heat pulses also play an important role in the entrainment of the molecular clock circuits to environmental temperature cycles.

PER and TIM proteins oscillate during temperature entrainment in constant darkness (dark/dark or DD), and these oscillations are maintained during constant conditions following entrainment [13]. It thus follows that temperature acts on at least some of the same molecular components of the circadian clock as light. Temperature cycles can also drive PER and TIM oscillations during constant light, a condition that, as mentioned earlier, normally results in behavioral and molecular arrhythmicity [17,23].

**Figure 1:**
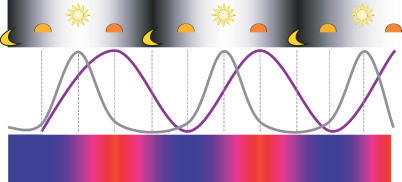
Relationship between Daily Light and Temperature Cycles Under natural environmental conditions, air temperature (purple line and blue = cold/red = warm scale) shows a more gradually changing profile than solar irradiance (gray line and black = dark/white = light scale). Incoming solar energy affects air temperature indirectly via the Earth's surface and this causes a lag between the profiles for sunlight and air temperature, with the temperature maximum and minimum occurring in the late afternoon and just before sunrise, respectively. These profiles are representative of calm, clear days; the lag in the environmental profile can be shortened or lengthened depending on factors such as cloud cover and wind (adapted from [25]).

In this report, we examine temperature as a *Zeitgeber* for the circadian clock and ask whether information from temperature is relayed through the same molecular circuits as light. In nature, the maxima and minima of solar irradiation and environmental temperature are offset (Figure 1) [25]. Sunrise generally coincides with the coolest part of the day and maximum solar irradiance at noon precedes the temperature maximum in the late afternoon. The divergent phases and waveforms of the environmental light and temperature profiles could in principle be represented by separate *Zeitgeber*-specific oscillators, or they could be integrated by a single oscillator capable of synchronizing to both photocycles and thermocycles.

By generating genome-wide transcriptional profiles during temperature cycles and subsequent constant conditions, we show that there are two distinct responses to temperature: a clock-independent, temperature-driven response and a clock-dependent, circadian response. Temperature-entrained circadian transcript profiles show a much higher degree of overlap with light-entrained circadian transcript profiles than do temperature-driven responses. Further, the mutual phase relationships among transcripts oscillating in response to both photo- and thermocycles are maintained in both conditions. Thus, many features of the circadian expression program emerge independently from the precise nature of the environmental *Zeitgeber.* Moreover, the molecular phases associated with separate photocycle and thermocycle entrainment suggest synergistic synchronization by the environmental light and temperature profiles found under most natural conditions.

## Results

### Genome-Wide Expression Profiles Indicate Two Distinct Responses to Temperature Entrainment

**Figure 2:**
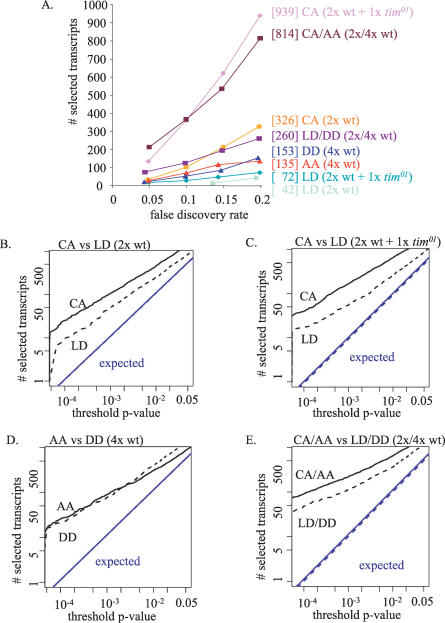
Comparison of Temperature-Driven, Light-Driven, Light-Entrained Circadian and Temperature-Entrained Circadian Daily Expression Programs The number of selected rhythmic transcripts for the indicated time course microarray datasets was determined as a function of the estimated FDR (A) or as a function of the threshold applied for the probability associated with 24-h spectral power (B–E). See Materials and Methods for a detailed description of the statistical procedures. Temperature-entrained and light-entrained circadian regulation are represented by 4-d wild-type (4x wt) datasets collected under constant conditions (25 °C in the dark) following temperature entrainment (AA) or light entrainment (DD) in (A) and (D), whereas temperature-driven and light-driven regulation are represented in (A) and (C) by datasets combining 2 d of wild-type plus 1 d of arrhythmic mutant data (2x wt + 1x *tim^01^*) collected in the presence of an 18 °C/25 °C thermocycle in the dark (CA) or in the presence of a 12-h light/12-h dark cycle at 25 °C (LD). In addition, datasets are included in (A) and (B) that consist of 2 d of wild-type CA or LD data representing a combination of temperature-driven and temperature-entrained circadian regulation or light-driven and light-entrained circadian regulation, respectively. Finally, 6-d wild-type datasets are considered in (A) and (E) that combine 2x CA and 4x AA or 2x LD and 4x DD data and represent temperature-entrained circadian regulation or light-entrained circadian regulation with some influence from temperature-driven or light-driven responses as well. Note that the y-axis scale in (A) is arithmetic but in (B–E) is geometric. The bracketed numbers in (A) indicate the number of selected transcripts for each of the analyses at FDR ∼0.2. Comparisons of the 24-h periodicity for these various datasets indicate that the circadian expression programs found in response to temperature or light entrainment have very similar properties, but that there is a large clock-independent temperature-driven expression program that clearly has a more global effect than circadian or light-driven regulation.

Genome-wide transcript profiles for the heads of temperature-entrained flies were determined in four 12-point time course experiments conducted in constant darkness; three time courses (two for wild-type and one for arrhythmic mutant *tim^01^* flies) consisting of a day-long 12-h 18 °C/12-h 25 °C (cold/ambient or CA) thermocycle plus a subsequent day of constant 25 °C (ambient/ambient or AA) and one time course (for wild-type flies) spanning the first two days of constant conditions (see Materials and Methods). In order to identify high-confidence, 24-h periodic gene expression, we compared the distribution of oscillatory statistics obtained from Affymetrix high-density oligonucleotide arrays to a permutation null model (see Materials and Methods; [26,27]). For each probe set on the arrays, we determined the 24-h spectral power and the probability of observing an equivalent or higher score from a genome-wide set of randomly permuted profiles. Analysis data are made available at http://biorhythm.rockefeller.edu. We then determined the number of selected 24-h periodic genes as a function of the threshold *p*-value or the associated false discovery rate (FDR) as illustrated by the graphs in Figure 2. These analyses were performed for various combinations of the new datasets representing temperature entrainment and of previously described datasets [27–29] representing light entrainment. Our analysis method emphasizes coherence of phase and period length but does not penalize inter-experimental variation in amplitude [26]. We have used this method to demonstrate that analysis of combinations of independently obtained time course microarray datasets representing the same or a similar environmental protocol allows for the detection of periodic expression programs with improved resolution [26,27]. The quantitative differences between the observed 24-h periodic expression programs are best visualized in Figure 2A, where an arithmetic scale is used.

It is clear that a 2-d wild-type dataset representing a CA environmental temperature cycle shows a much broader impact on global 24-h periodicity than an equivalent dataset representing a 12-h light/12-h dark (LD) cycle (e.g., 326 versus 42 periodic transcript profiles at FDR 0.2; see Figure 2A and 2B). This result could be explained by either a temperature-driven, clock-independent effect or by a thermocycle-specific, clock-dependent effect. Additional comparative analyses help to distinguish between these two possibilities. First, consider expanded 3-d versions of the CA and LD datasets that each also include a 1-d time course in the same format obtained from arrhythmic *tim^01^* flies. Given that both behavioral and molecular circadian rhythms appear to be abrogated by the *tim^01^* mutation [27,30], the additional time course data may represent temperature- or light-driven, but not clock-dependent, expression profiles. The enhanced difference in 24-h periodicity between thermocycle and photocycle conditions that results from inclusion of the *tim^01^* data (e.g., 939 versus 72 periodic transcript profiles at FDR 0.2; Figure 2A and 2C) indicates that most of the thermocycle-associated transcript rhythms are simply temperature-driven independently from the clock. Second, compare the properties of 24-h periodic transcript profiles of 4-d datasets representing the same constant conditions (25 °C and constant darkness) after either temperature entrainment (AA) or light entrainment (DD). The circadian programs detected after entrainment to temperature and light have very similar properties (Figure 2A and 2D; see also the section Defining a Set of Clock-Dependent Transcripts, below), supporting the hypothesis that the increased 24-h periodicity found in the context of an environmental temperature cycle is due to clock-independent temperature-driven regulation rather than to circadian rhythms that specifically require temperature entrainment.

The broad temperature-driven response observed in the presence of a temperature cycle could in principle interfere with the circadian expression program. This issue is addressed by analysis of a 6-d dataset combining 2 d of temperature entrainment and 4 d of subsequent constant conditions (CA/AA) and an equivalent 6-d dataset representing light entrainment and subsequent constant conditions (LD/DD). Both of these programs indicate more high-quality daily transcript oscillations than are found separately for the 2-d (CA or LD) or 4-d (AA or DD) subsets (Figure 2A and 2C–2E). Even the sum total of high-quality daily transcript oscillations from the 2-d and 4-d subsets is considerably less than the number observed for the integrated 6-d sets (e.g., 33 for 2x CA plus 27 for 4x AA versus 212 for 2x CA/4x AA and 13 for 2x LD plus 20 for 4x DD versus 75 for 2x LD/4x DD at FDR 0.05, Figure 2A and 2C–2E). This suggests that, in general, circadian expression profiles are not dramatically altered by temperature-driven (or light-driven) effects (Figure 2A, 2B, 2D, and 2E).

Comparison of the 24-h periodicity found in the 6-d wild-type CA/AA and LD/DD sets versus the 4-d wild-type AA and DD sets and the 3-d wild-type plus *tim^01^* CA and LD sets further illustrates the magnitude of the temperature-driven response (Figure 2A, 2C, and 2E). There is no reason to assume that purely circadian expression profiles are more prevalent or prominent in CA versus LD conditions, yet inclusion of 2 d of CA data with the AA set has a bigger impact on 24-h periodicity than the inclusion of 2 d of LD data with the DD set, suggesting that temperature-driven regulation may be responsible. In addition, although more extensive 24-h periodic expression is generally detected for larger datasets [26,27], the daily expression program found for the 3-d wild-type plus *tim^01^* CA dataset is comparable in size to that for the 6-d wild-type CA/AA dataset. In contrast, the 6-d wild-type LD/DD dataset shows much more extensive 24-h periodicity than the 3-d wild-type plus *tim^01^* LD dataset, indicating that the presence of a daily thermocycle is the primary determinant of the number of observed 24-h transcript oscillations.

Taken together, these analyses suggest that the circadian expression programs entrained by light entrainment and temperature entrainment have similar properties, whereas an environmental thermocycle directly evokes a global expression response that is considerably broader than that found for light-dependent or clock-dependent regulation.

### Defining a Set of Temperature-Driven Transcripts

**Figure 3:**
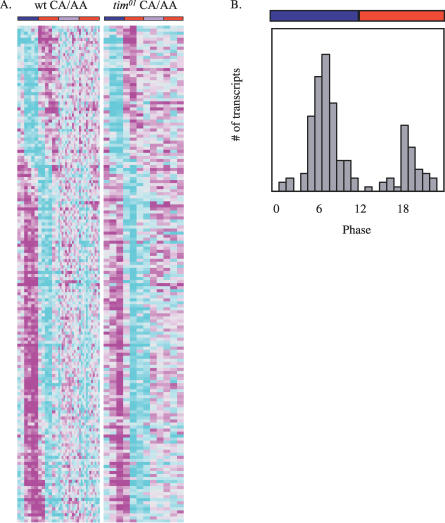
Phases of the Temperature-Driven Transcripts (A) Phasegrams for transcripts from wild-type (wt) and *tim^01^* flies in CA/AA are shown. Columns correspond to time points, and transcript profiles are represented by rows. Rows are ordered according to the estimated peak phase of the transcript profiles in CA conditions. Expression values represented by increasingly bright shades of magenta and cyan indicate, respectively, upregulation and downregulation relative to the experimental average (indicated by light gray). (B) Histogram showing the estimated peak phases (ZT h) of the temperature-driven transcripts. The red, blue, and violet bars in (A) and (B) indicate the warm, cold, and subjective cold phases, respectively.

A core set of the most robustly temperature-driven transcripts was identified based on wild-type and *tim^01^* thermocycle expression profile data. In order to be included, transcripts had to meet several noise filters and show a highly significant 24-h Fourier component as well as a significant 24-h autocorrelation (see Materials and Methods). The phasegram for the resulting set of 164 temperature-driven transcripts in both wild-type and *tim^01^* flies is shown in Figure 3A. The majority of these transcripts respond with a simple pattern of either activation during the warm phase and repression during the cold phase or vice versa. Further, most of this response is lost in constant conditions following temperature entrainment (Figure 3A) or during and after light entrainment (unpublished data), emphasizing that the response is driven and not circadian.

Figure 3B shows the average peak phases of the temperature-driven transcripts across data from wild-type and *tim^01^* flies during temperature entrainment. Two trends are obvious from this analysis: (1) temperature-driven oscillations tend to peak around the middle of either the cryophase or the thermophase, and (2) more transcripts peak during the cryophase than during the thermophase. If the term CA0 is assigned to the onset of the cold temperature and CA12 to the onset of the ambient temperature, the majority of the transcripts have a phase of either CA5–8 (toward the middle of the cryophase) or CA18–20 (toward the middle of the thermophase). Given our use of sine wave fits to estimate peak phases, the observed phase distribution is consistent with a majority of the expression profiles being directly positively or negatively temperature-driven with relatively little delay. Approximately three-quarters of the temperature-driven profiles peak in the cryophase, but the functional relevance of this preference is not directly obvious.

**Figure 4:**
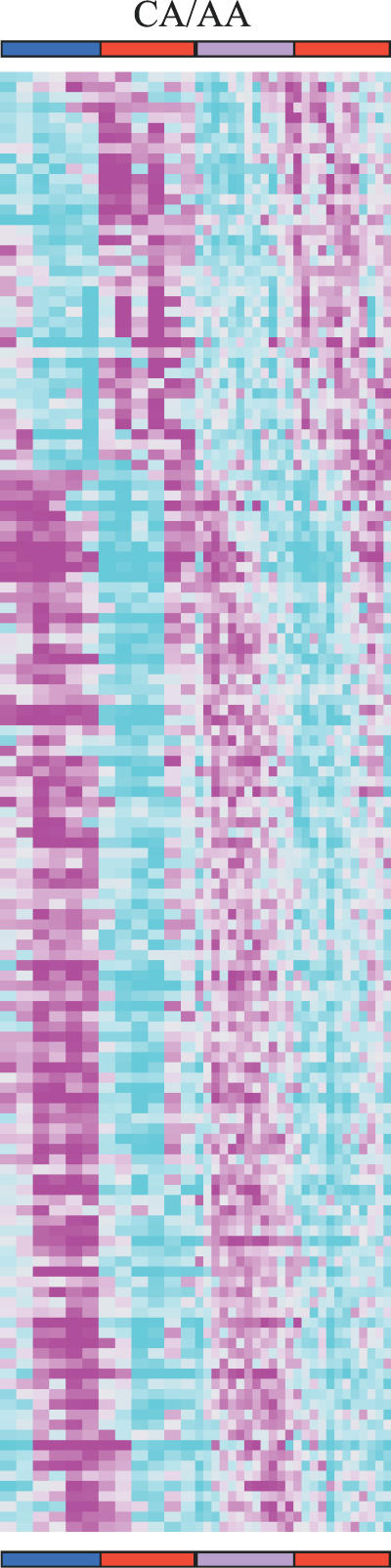
Phases of the Clock-Dependent Transcripts The transcripts from wild-type flies in CA/AA are shown by phase in the same format as Figure 3. Columns correspond to time points, and transcript profiles are represented by rows. Rows are ordered according to the estimated peak phase of the transcript profiles across the CA/AA data. Expression values represented by increasingly bright shades of magenta and cyan indicate, respectively, upregulation and downregulation relative to the experimental average (indicated by light gray). The red, blue, and violet bars above the phasegram indicate the warm, cold, and subjective cold phases, respectively.

The temperature-driven transcripts are representative of diverse biological functions (Table S1), including carbohydrate, amino acid, lipid/fatty acid, one-carbon compound, nucleic acid, folate, and steroid metabolism, as well as transport, signal transduction, development, behavior, protein translation, protein modification, protein folding, proteolysis, defense/immune response, muscle contraction, cytoskeleton, and exoskeleton. In comparison with a set of 143 predicted temperature-entrained circadian transcripts that is described in more detail below (see Figure 4 and Materials and Methods), the temperature-driven transcripts show a higher frequency of functions associated with transport, transcription, translation, development, proteolysis, and protein folding, but a lower frequency of functions associated with circadian behavior and carbohydrate metabolism.

### Defining a Set of Clock-Dependent Transcripts

**Figure 5:**
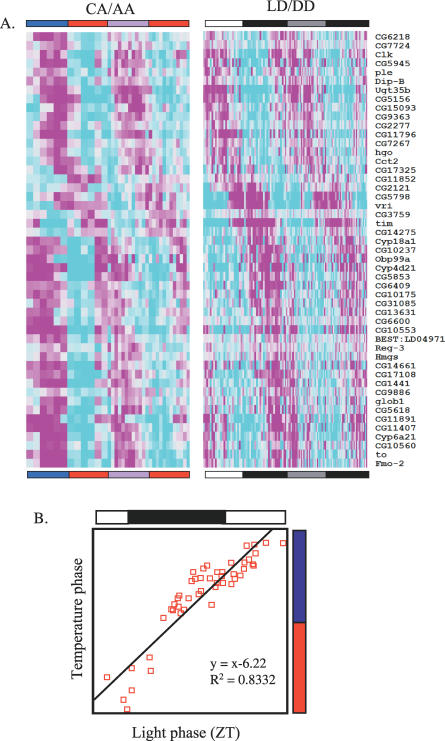
Overlap and Mutual Phase Relationship between Those Transcripts Oscillating in CA/AA and LD/DD (A) Wild-type transcripts oscillating in response to both photo- and thermocycles are shown by phase in the same format as Figure 3. Columns correspond to time points, and transcript profiles (with gene names listed to the right) are represented by rows. Rows are ordered according to the estimated peak phase of the transcript profiles across the LD/DD data. Expression values represented by increasingly bright shades of magenta and cyan indicate, respectively, upregulation and downregulation relative to the experimental average (indicated by light gray). The red, blue, violet, white, black, and gray bars above the phasegram indicate the warm, cold, subjective cold, light, dark, and subjective light phases, respectively. (B) The phases of transcripts oscillating in CA/AA are “advanced” (relative to the onset of the respective *Zeitgeber*) by about 6 h as compared to LD/DD. The bars above and to the right of the plot denote the entrainment scheme. Each red square on the plot corresponds to a transcript, with its LD/DD phase indicated on the x-axis and its CA/AA phase indicated on the y-axis. The data were fit to a regression line with slope 1 as indicated.

**Figure 6:**
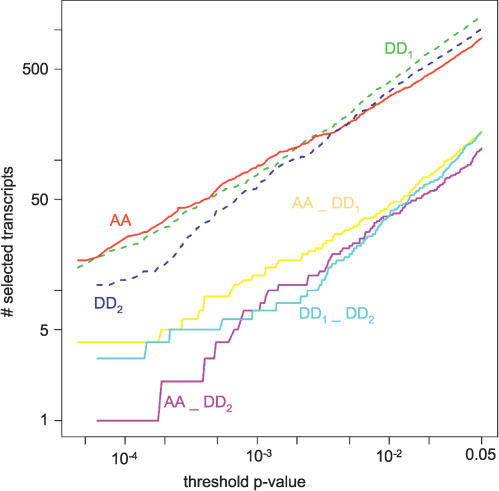
Overlap Analysis of Independently Determined Circadian Expression Programs after Light or Temperature Entrainment Three independent 4-d wild-type (4x wt) datasets collected under constant conditions (25 °C in the dark) following temperature entrainment (AA) or light entrainment (DD_1_ and DD_2_) are indicated. See Materials and Methods for details. The number of selected rhythmic transcripts for each of these datasets is graphed as a function of the threshold applied for the probability associated with 24-h spectral power (upper three lines). The size of the pairwise overlap between the circadian transcript selections as a function of the selective *p*-value is also indicated (lower three lines).

All available microarray time courses from wild-type flies during and after temperature entrainment were used to define a set of temperature-entrained circadian transcripts. In order to be included, transcripts had to meet several noise filters and show a highly significant 24-h Fourier component as well as significant 24-h autocorrelation (see Materials and Methods) across the complete 2x CA/4x AA dataset. In order to avoid transcript oscillations that were merely temperature-driven, it was required that they also show a significant 24-h Fourier component and exceed background noise in an analysis of AA data only (see Materials and Methods). The resulting set of 143 temperature-entrained circadian transcripts is presented in a phasegram (Figure 4), and the functions associated with these transcripts are described in Table S1. As noted above, the set of temperature-entrained circadian transcripts shows some differences in its functional representation relative to the set of temperature-driven transcripts. Perhaps the most remarkable functional enrichment among temperature-entrained circadian transcripts is found for the *takeout (to)* gene family, which has been proposed to contribute to courtship behavior, starvation response, and olfaction [31,32]. Seven of the 21 members of this gene family show a strong temperature-entrained circadian expression component, whereas a robust temperature-driven response (represented by oscillating expression in a temperature cycle in *tim^01^* flies) is only found for one gene, which happens to also show circadian regulation (Table S1). We also defined a set of 172 light-entrained circadian transcripts by applying similar selection criteria to the results from previous analyses of all available LD/DD microarray time course data [27] (see Materials and Methods). The overlap between the two datasets (49 transcripts) is highly significant and involves about a third of the transcripts in each set (Figure 5A and Table S1), which is considerably more than, for example, the overlap of either set with the set of 164 temperature-driven transcripts from Figure 4 (overlap of 22 with the temperature-entrained and 13 with the light-entrained circadian transcripts). The fact that two-thirds of the predicted temperature-entrained circadian transcript profiles are not represented in a stringently selected set of light-entrained circadian transcript profiles does not mean that they do not show significant light-entrained circadian oscillations. The degree of overlap between the independent selections is quite sensitive to factors such as choice of selective cut-off criteria and reproducibility of the relative rankings of circadian transcripts. Nevertheless, the fact that the overlap is incomplete could indicate that there are both light- and temperature-specific circadian transcript oscillations. To examine the relative importance of such *Zeitgeber*-specific entrainment, we tested whether the overlap between two independent 4-d DD datasets was substantially larger than that between each of the two DD sets and our 4-d AA dataset; the results are illustrated in Figure 6. The two independently detected DD circadian expression programs did not obviously share more transcripts with each other than with the AA circadian expression program. There is, therefore, no evidence for widespread temperature-specific or light-specific circadian expression. To further investigate this issue, six potential temperature-specific and three potential light-specific circadian transcript profiles were verified using northern blots. All six of the potential temperature-specific circadian transcripts show circadian oscillations in LD/DD (Figure S1A), whereas none of the three transcripts with a predicted light-dependent circadian response exhibited clock-dependent regulation in response to temperature entrainment (Figure S1B). These results are consistent with the notion that most, but not all, light-entrained circadian transcripts are also entrained by an environmental temperature cycle.

### Environmental Photocycles and Thermocycles Entrain the Circadian Clock to Similar Molecular and Behavioral Phases

Identification of a set of transcripts that show especially robust circadian regulation in response to both entrainment by light and entrainment by temperature allowed us to directly compare the circadian phases dictated by these two *Zeitgebers.* When all available LD/DD and CA/AA time course microarray data for this set is ordered in a phasegram according to the observed light-entrained phase, it becomes apparent that temperature-entrained phases are directly correlated with independently determined light-entrained phases, suggesting that mutual phase relationships among circadian transcripts may be preserved under entrainment of either light or temperature (Figure 5A). When estimated light-entrained and temperature-entrained peak phases are directly plotted against each other, it becomes apparent that their relationship can be described by a linear function that represents a fixed phase offset (Figure 5B). When the light-entrained phase is determined relative to lights-on and the temperature-entrained phase is determined relative to the onset of the thermophase, the value of the offset is ∼6 h. In the context of natural environmental conditions (Figure 1), however, the light/dark and temperature cycles are aligned differently, with the temperature minimum occurring just before dawn and the temperature maximum delayed relative to the time of maximum solar irradiance. It would be, therefore, more appropriate to assign temperature-entrained phases relative to the time of subjective dawn (the middle of the cryophase). In this context, the observed temperature-entrained and light-entrained molecular phases would, in fact, roughly coincide, indicating cooperative entrainment by environmental cycles in light and temperature under most natural conditions. Convergence of photic and thermal entrainment is also observed at the behavioral level (Figure S2). During LD cycles, flies are preferentially active around the dark-to-light and light-to-dark transitions, with a siesta in the middle of the day. Although the sudden changes in environmental light at the transitions directly elicit behavioral startle responses in a clock-independent manner, a functional clock is required for control of a major circadian activity component at dusk and a minor circadian activity component at dawn (e.g., [18]). During temperature entrainment, the outlines of a similar pattern of clock-dependent activity can be recognized with onset of dawn-associated activity occurring during the last half of the cryophase and dusk-associated activity coinciding with the middle of the thermophase (Figure S2).

**Figure 7:**
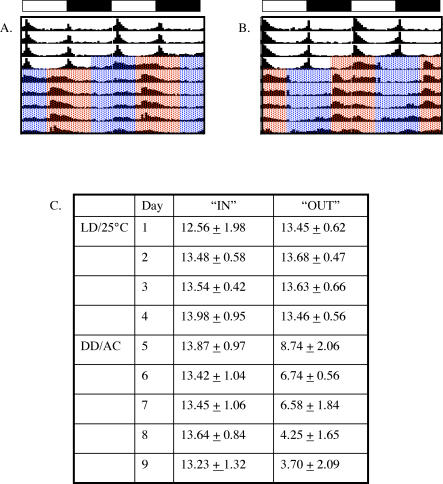
Cooperative and Antagonistic Effects of Light and Temperature on Locomotor Activity Behavior (A–B) The average locomotor activity of a number of flies (A, *n* = 23; B, *n* = 18) is represented in each panel. The data are double plotted for visual continuity. Flies were recorded for 4 d in an LD cycle, as indicated by the open and closed bars above the panels, respectively. The flies were then released into DD in which a 25 °C/18 °C temperature cycle was given “in” phase (A, onset of light precedes the onset of warm temperature by 6 h) or “out” of phase (B, onset of warm temperature precedes the onset of light by 6 h). (C) The average times of activity offset relative to the initial LD cycle and the associated standard deviation are indicated on each day for flies with in-phase or out-of-phase thermocycles.

We further examined the cooperative effects of light and temperature on entrainment of locomotor activity behavior. We entrained flies to an LD cycle and then released them into constant darkness in which a temperature cycle was given either “in phase” (i.e., light onset precedes onset of the thermophase by 6 h) or “out of phase” (i.e., thermophase onset precedes light onset by 6 h). When light and temperature are given in phase, the average time of activity offset after 5 d of temperature is the same as the previous light phase (*Zeitgeiber* time [ZT] 13.23 ± 1.32 versus ZT 13.98 ± 0.95; Figure 7). However, when light and temperature are given out of phase, the average time of activity offset gradually advances over 5 d of temperature to approximately 10 h earlier than that measured in LD (ZT 3.70 ± 2.90 versus ZT 13.46 ± 0.56; Figure 7B and 7C). Thus, the temperature- and light-entrained circadian behavioral phases show essentially the same relationship as the molecular phases that we discussed above. Under “natural” environmental conditions (i.e., when light onset and offset coincide with the middle of, respectively, the cryophase and thermophase) both molecular and behavioral rhythms are entrained cooperatively by temperature and light.

### Organization of the Core Clock Components in Temperature Entrainment

**Figure 8:**
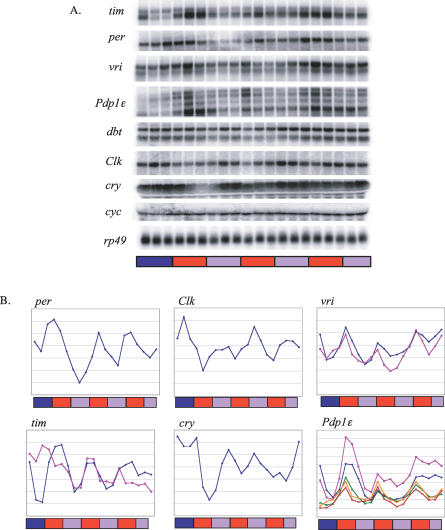
Expression of the Core Clock Genes in Temperature Entrainment (A) Northern blots showing expression of the core clock transcripts in wild-type flies in CA and AA. An *rp49*-specific probe was used as a loading control. (B) Quantitations from (A). The bars below the northern images and plots denote the entrainment scheme, with red bars indicating 25 °C time points, blue bars indicating 18 °C time points, and violet bars indicating free-run time points taken at 25 °C. The different colored lines in the *vri, tim,* and *Pdp1* plots represent the different transcripts. At least two independent profiles were obtained for each transcript. Peak to trough ratios (P/T) across the entire experiment, probability of circadian rhythmicity (pF24), and predicted circadian phase relative to the onset of the cryophase (phF24) are as follows. *per* (P/T = 4.8; pF24 = 0.02; phF24 = CA16), *tim* (smaller transcript P/T = 5.8; pF24 = 0.0001; phF24 = CA18; larger transcript [*tim^cold^*] (P/T = 2.7; pF24 = 0.04; phF24 = CA15), Clk (P/T = 2.7; pF24 = 0.0001; phF24 = CA3), cry (P/T = 4.3; pF24 = 0.04; phF24 = CA8), *vri* (larger transcript P/T = 2.7; pF24 = 0.15; phF24 = CA13; smaller transcript P/T = 2.2; pF24 = 0.12; phF24 = CA19), *Pdp1* (transcripts numbered according to increasing size, *Pdp1-1* [not visible on blot] P/T = 3.3; pF24 = 0.18; phF24 = CA21, *Pdp1-2* P/T = 4.6; pF24 = 0.02; phF24 = CA20, *Pdp1-3* P/T = 3.3; pF24 = 0.003; phF24 = CA17, *Pdp1-4* P/T = 3.2; pF24 = 0.0003; phF24 = CA15, *Pdp1-5* P/T = 3.4; pF24 = 0.003; phF24 = CA15).

The well-characterized core clock transcripts that are known to oscillate under light entrainment *(per, tim, Clk, cry, vri, Pdp1ɛ)* continue to cycle under temperature entrainment in wild-type flies, while the noncycling core clock transcripts *(cyc, dbt)* remain constant (Figure 8). In LD cycles, the levels of the core clock transcripts remain at constitutive high or low levels in arrhythmic *tim^01^* flies [4,30,33–35]. Analysis of the *tim, per, Clk,* and *cry* transcripts in arrhythmic *tim^01^* flies exposed to a temperature cycle, however, reveals underlying temperature-driven responses that do not persist under constant conditions (Figure S3). These temperature-driven oscillations are mostly out of phase with the circadian transcript rhythms observed in wild-type flies (Figure S3). In order to maintain essentially the same circadian expression program for its core components in the presence or absence of an environmental temperature cycle, therefore, the clock has to largely neutralize the direct effects of temperature on their expression.

The phase relationships between core clock transcripts (i.e., *per, tim, vri,* and *Pdp1ɛ* oscillate antiphase to *Clk* and *cry*) are largely maintained in wild-type, temperature-entrained flies. Entrainment to temperature cycles, therefore, appears to promote the same overall clock organization and function as light entrainment, although with an important distinction that involves *per* and *tim* RNA expression. In light entrainment and subsequent free-run, *per* and *tim* transcription is tightly coupled at all times (Figure S4). In temperature entrainment, however, *per* and *tim* are uncoupled. This is due to both an advance in *per* expression and a delay in *tim* expression (compared to free-run; Figure S4). This divergence is absent in constant conditions following temperature entrainment. It is also absent at the protein level (Figure S5). One possible explanation for this discrepancy is that in temperature cycles a shift in the timing of *per* and *tim* RNA accumulation may be required to maintain coordinately phased accrual of the PER and TIM proteins.

**Figure 9:**
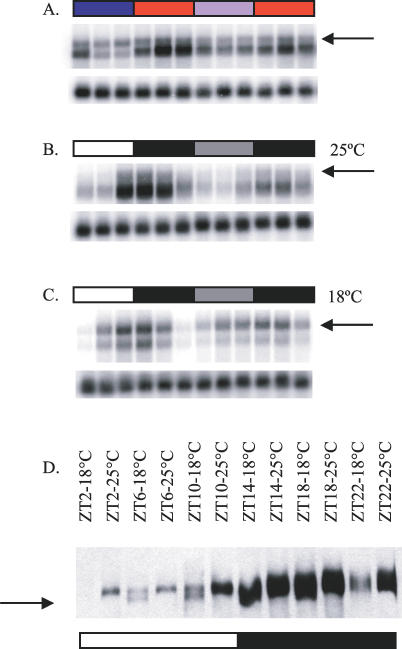
*tim* Is Alternatively Spliced at Cold Temperatures (A–C) An alternatively spliced form of *tim* RNA (arrows in A–C) is present in wild-type flies in CA/AA (A), especially during the cold phase, and in LD/DD at 18 °C (C). This splice form is less abundant and distinct in flies entrained to LD/DD at 25 °C (B). An *rp49*-specific probe was used as a loading control for each blot (lower panels). (D) The alternative transcript contains a predicted premature stop codon and results in a shorter TIM protein isoform (arrow), which can be readily detected in samples collected in LD at 18 °C but is not obvious at 25°C. The horizontal color-coded bars in panels in (A–D) denote the entrainment scheme, with white bars indicating light time points, black bars indicating dark time points, gray bars indicating free-run time points taken during subjective light, red bars indicating 25 °C time points, the blue bar indicating 18 °C time points, and the violet bar indicating free-run time points taken at 25 °C.

The advance in *per* expression could in theory be explained by the effects of a thermosensitive splicing event in the 3′ UTR of *per,* which is thought to enable flies to seasonally adapt to cold, short days [36–38]. It is important, however, to address this hypothesis experimentally, as alternative splicing of *per* has not been directly examined in the context of a thermocycle. The delay in *tim* expression is associated with thermosensitive splicing. A second *tim* transcript (referred to as *tim^cold^*) is observed during temperature entrainment, especially during the cold phase (Figure 9A). *tim^cold^* is expressed at low levels in light entrainment at 25 °C (Figure 9B), but in light entrainment performed at 18 °C it is the dominant isoform (Figure 9C). Overall *tim* transcript levels appear to be increased at 18 °C relative to 25 °C by a factor of 1.5–2 (unpublished data).While the canonical shorter transcript of *tim* oscillates with a phase that differs from that of *per* during temperature entrainment, *tim^cold^* cycles in phase with *per* (Figure S4). Total *tim* transcript levels in the presence of the 18 °C /25 °C thermocycle follow the same pattern as the canonical transcript, albeit at a somewhat lower peak/trough ratio (∼3-fold versus ∼5-fold). Reverse transcriptase-PCR and northern blot analyses reveal that, in *tim^cold^,* the last *tim* intron (∼850 bp) is retained (unpublished data). This unspliced form of *tim* has a premature stop codon that would putatively result in a protein about 3.5 kDa smaller than the full-length TIM protein. The missing fragment corresponds to a small piece of the cytoplasmic localization domain [39]. Western blots reveal the presence of two TIM isoforms at 18 °C in light entrainment, the lower of which is downregulated at 25 °C (Figure 9D). Further work will be needed to understand the role of *tim* alternative splicing.

## Discussion

**Figure 10:**
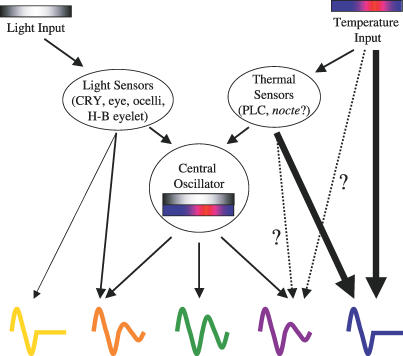
Model of How Information from Light and Temperature Are Processed by the Fly Circadian Clock Information from light and temperature, which is naturally out of phase, is relayed through the appropriate sensors to the clock. In the absence of photic or thermal input, the clock can predict when the fly would have seen light and dark or warm and cold, respectively. When both *Zeitgebers* are present, they are integrated by the clock to generate meaningful phases of transcription (green). Independently of the clock, light can directly affect (through light sensors) the transcription of a small number of genes (yellow), whereas temperature can drive the expression of a larger number of transcripts (blue). Although some potentially relevant thermal sensors have been reported based on genetic evidence [17], it is unclear to what extent they are involved in determining temperature-entrained and temperature-driven transcript rhythms at a genome-wide level. There are also transcripts that are dually regulated by the clock and light (orange) or temperature (purple), which may be important for processes such as seasonal adaptation.

Light is the best understood *Zeitgeber* for the circadian clock, but many organisms are also exposed to daily changes in temperature in their natural environments that may influence their clocks. In this study, we describe entrainment of molecular and behavioral circadian rhythms by environmental thermocycles. Genome-wide expression profiles of transcripts during temperature entrainment and subsequent constant conditions in both wild-type and arrhythmic *tim^01^* backgrounds were generated in order to analyze the role of temperature cycles on gene expression in the fly. Unlike in light entrainment, where the magnitude of the overall transcriptional response in the presence or absence of a photocycle is largely maintained, there is a dramatic difference in transcriptional responses in the presence or absence of a thermocycle. Our results indicate, therefore, that temperature-driven responses may be a major determinant of daily fluctuations in gene expression. Because the day-to-day variability in temperature profiles found in most natural climates would negatively impact the stability of temperature-driven daily expression rhythms, the observed temperature-driven regulation of gene expression may primarily serve to mediate short-term responses to changes in temperature. Whereas transcription appears to be modified globally by temperature cycles, there are a limited number of transcripts that continue to oscillate in constant conditions following temperature entrainment. Thus, temperature cycles elicit both a clock-independent, temperature-driven response and a clock-dependent, circadian response (Figure 10).

A set of clock-independent, temperature-driven transcript profiles was defined by analyzing both the wild-type and *tim^01^* data in the presence of a thermocycle. Since these transcripts are directly driven by a thermocycle, their behavior should be the same in the presence or absence of a functional clock. However, due to the inclusion of wild-type data, this set could, in theory, include transcripts that are both temperature-driven as well as clock-dependent. Dual regulation by both the circadian clock and temperature or light may allow for seasonally modulated daily transcript rhythms. A set of dually light- and clock-regulated transcripts was recently identified in the context of an LD cycle [27] (Figure 10). In an arrhythmic clock mutant, these transcripts are simply induced or repressed in response to light. In wild-type flies, however, input from both light and the clock results in transcript profiles combining light-driven and circadian expression components. In the present study, we found significant overlap between the set of transcripts that show temperature-driven regulation and the set of either temperature-entrained or light-entrained circadian transcripts. For example, 22 transcripts are part of both the temperature-driven set described in Figure 3 and the temperature-entrained circadian set described in Figure 4, indicating that there is, indeed, a class of transcripts with both circadian- and temperature-driven regulation, whose circadian- and temperature-driven expression components are well aligned. Moreover, our in-depth analysis of core clock transcript profiles has uncovered a second class of dually temperature- and clock-regulated transcripts, whose circadian- and temperature-driven expression components favor opposite peak expression phases. Comprehensive identification of transcripts belonging to this second class would require collection of additional time course microarray data, so that thermocycle-associated periodicity and phase can be determined separately for arrhythmic mutant flies.

We also defined a set of clock-dependent, circadian transcripts in this study. This set of temperature-entrained transcripts shows a highly significant overlap with those transcripts that oscillate in response to a photocycle. Further, the light- and temperature-entrained phases of these transcripts roughly coincide in the context of natural environmental conditions. These observations indicate that in the fruit fly a single TIM-dependent transcriptional clock mechanism produces a core circadian expression program that can be synchronized to different environmental *Zeitgebers.* Although the global properties of the light-entrained and temperature-entrained circadian expression programs are very similar, we have identified three examples of genes that only exhibited circadian regulation in response to an environmental light/dark cycle. This novel type of regulation may represent a previously unrecognized functional interaction between light-sensing pathways and the circadian clock. It is also possible, however, that the apparently light-specific circadian genes are not completely insensitive to temperature entrainment, but merely require higher thermocycle amplitude or a prolonged period of entrainment.

### Differential Regulation of *per* and *tim*

Transcriptional regulation of *per* and *tim* appears to be different in light and temperature entrainment. Whereas in light entrainment *per* and *tim* RNA expression is tightly coupled at all times, in 18 °C/25 °C temperature entrainment *per* RNA levels peak before *tim* RNA levels. This is a result of a temperature-induced advance in *per* expression and delay in the expression of the predominant *tim* transcript. Differences in *per* and *tim* regulation have been suggested based on the observation that these transcripts show different rates of degradation in response to a light pulse in the context of the long period mutant *tim^ul^* [40]. In addition, while at lower temperatures *per* expression is upregulated in LD and DD, *tim* has been reported to be downregulated in LD and barely oscillatory in DD [38]. Further, while the phases of both *per* and *tim* appeared advanced at lower temperatures, the advance in *per* was interpreted as a result of faster accumulation, while the advance in *tim* was thought to represent more rapid degradation [38]. It has also very recently been reported that *tim,* but not *per,* transcript levels are upregulated in response to light pulses at cold temperatures [41]. It is noteworthy, however, that the probe used in several previous studies [24,38,41] to evaluate *tim* transcript expression with RNase protection assays may not have efficiently detected the *tim^cold^* isoform since it spans the exons flanking the intron maintained in *tim^cold^.* This issue is illustrated by quantitation of the data in Figure 9C, which confirms the predicted decrease of *tim* transcript in the first day of DD at 18 °C [38] for the predominant isoform, but not for *tim^cold^,* which shows a more prominent peak in expression (unpublished data). Additional analyses that take into account the contribution of the *tim^cold^* isoform will, therefore, be needed to complement previous studies in order to more fully explore *tim* transcript responses.

One of the factors involved in the reported differential expression of *per* and *tim* may be the alternative splicing of both transcripts. Much of the recent molecular work on temperature and the circadian clock has focused on the alternative splicing of an 89-bp intron in the 3′ UTR of *per,* an event thought to be important in seasonal adaptation [36–38]. Short, cold days lead to increased amounts of the spliced *per* variant, resulting in an earlier increase in PER protein abundance and an advanced phase of locomotor activity. Warmer temperatures result in less of the spliced variant, especially during the day. This appears to be a clock-dependent effect that results in the fly moving its behavior to the later (cooler) part of the day. Thus, *per* splicing allows the fly to adapt to changes in both temperature and photoperiod by regulating the amount of available PER protein. *per* alternative splicing is thought to be important in seasonal adaptation, as long photoperiods counteract the cold-induced behavioral advances by delaying the accumulation of TIM, in turn rendering prematurely produced PER unstable. Thus, the fly is able to integrate information from both light and temperature to generate behavior that is aligned to the environmental day. Regulation of *per* splicing in the presence of an environmental temperature cycle as compared to constant temperature needs to be investigated.

Temperature-dependent alternative splicing of *tim* is described here. At 18 °C, the last intron of *tim* is preferentially retained, resulting in a premature stop codon and a truncated protein. Although the expression of the predominant *tim* transcript is delayed relative to *per, tim^cold^* cycles in phase with *per.* The differential expression of the two *tim* transcripts could reflect temperature-dependent control of splicing or of the stability of one of the splice forms. We are still in the process of ascertaining the functional significance of the production of *tim^cold^* transcript. It does, however, appear that the alternative splicing and differential regulation of *per* and *tim* are responsible for finely tuning the clock in response to changing environmental conditions, thus adding an additional level of complexity to the clock.

### Two *Zeitgebers,* One Clock

Different groups of clock-bearing cells in the fly have been shown to regulate different rhythmic processes. For example, locomotor activity and eclosion rhythms, arguably the best-characterized rhythmic behaviors in *Drosophila,* require the ventral lateral neurons (LN_v_s) and the neuropeptide, Pigment Dispersing Factor [42–44]. Cyclic olfactory responses do not depend on the LNs or Pigment Dispersing Factor, but instead depend on the antennal neurons [45,46]. Egg-laying rhythms also appear to be regulated independently of the LN_v_s and Pigment Dispersing Factor [47]. Thus, the image of the circadian clock as a single entity is transforming into a more complex model.

A system of two coupled oscillators was proposed for the *Drosophila* clock almost 50 y ago [48]. In this model, the master or A oscillator is autonomous, light-sensitive, and temperature-compensated. The slave or B oscillator, which is coupled to and driven by A, is responsive to temperature but not light. The evidence for this two-oscillator model came from the different responses in eclosion rhythms to light and temperature. Whereas light pulses administered at different times of day resulted in steady-state phase advances or delays, the phase changes resulting from temperature pulses were transient. The researchers concluded that the steady-state phase changes in response to light were a result of the eventual realignment of the A oscillator to the light signal. The transient responses to temperature pulses were proposed to be a result of temporary temperature-induced disturbances in B, with the return to the previous phase reflecting the A oscillator's resumption of control over B.

Coupled oscillators have been proposed in a variety of organisms [49]. For example, different genes are expressed with different period lengths in some *Synechoccus* mutant backgrounds [50], and bioluminescence rhythms in *Gonyaulax* have been shown to be regulated by two oscillators that respond to different wavelengths of light [51]. In *Neurospora,* strains carrying null alleles of *frequency (frq), white collar-1 (wc-1),* or *white collar-2 (wc-2)* still show a conidial banding rhythm. Although the “FRQ-less oscillators” [52] responsible for these rhythms have lost most characteristics of a circadian clock, they can be entrained by *Zeitgebers* such as temperature cycles [53,54], rhythms in nitrate reductase activity [55], and transfer from light to dark [56]. This suggests that FRQ-less oscillators are slave oscillators, requiring the master FRQ-dependent clock to produce stable circadian rhythms, yet with the ability to function independently of the master clock in a non-circadian manner [57].

A system of coupled oscillators has recently been demonstrated in the regulation of the morning and evening peaks of locomotor activity in the fly [58–60]. The morning oscillator requires the presence of the LN_v_s, while the evening oscillator requires the dorsal lateral neurons. It was further shown that the evening oscillator is set by the morning oscillator by generating flies in which the morning and evening oscillators have different free-running periods [60]. However, despite the parallels to Pittendrigh's original model, there is no published evidence that these or other oscillators would differentially respond to temperature, as opposed to light, as a *Zeitgeber.* So while it appears there is a multicellular clock network in *Drosophila* that is reflected by coordinate yet independently regulated outputs, the data presented here suggest that the response to multiple inputs, such as light and temperature, would still be integrated by a single autonomous clock mechanism (Figure 10). In today's jargon we would describe Pittendrigh's B oscillator as a circadian output pathway that can show direct clock-independent responses to temperature.

The following observations support the hypothesis of a single, integrative transcriptional oscillator. First, the same set of core clock components (including PER, TIM, CLK, and CYC) appears to be required for producing both light-entrained and temperature-entrained oscillations [18,19]. The global transcriptional signatures of arrhythmic *tim^01^* flies that we found after thermocycle treatment resemble those found after photocycle treatment [27] and do not exhibit obvious circadian rhythms. In addition, our results confirm the absence of circadian oscillations for core clock gene transcripts in the *tim^01^* fly heads. Second, it is likely that the set of transcripts entrainable by thermocycles is closely related to the set of transcripts entrainable by light. Although we cannot formally exclude the existence of circadian rhythms that specifically require temperature entrainment, we have found none so far. Third, the phases of the transcripts that oscillate in response to both photo- and thermocycles maintain the same mutual phase relationships after entrainment by light or temperature. The phase observed at the onset of the thermophase is systematically advanced by about 6 h relative to the phase at the onset of light. Given the size of the delay that is commonly found between the environmental profiles for temperature relative to that of daylight, these results indicate cooperative entrainment by light and temperature under common natural circumstances. A response to temperature would be well integrated with the expected light cycle were it also supplied, and vice versa. Fourth, the temperature- and light-entrained phases of PER and TIM protein expression [13] (see also Figure S5) reflect the same relationship that we observed for the genome-wide circadian transcript signatures. This observation is consistent with the hypothesis that both light and temperature act via the same PER/TIM-dependent oscillator to generate circadian transcript profiles. Fifth, the entrained phase of locomotor activity behavior appears to follow the molecular circadian phase observed in temperature or light entrainment. Our ability to accurately predict the phase of clock neuron–controlled circadian locomotor behavior based on our analysis of circadian transcript rhythms in a preparation of whole heads, which mostly represents peripheral clock cells, suggests that temperature entrainment just as light entrainment produces similar phases in peripheral clock cells and clock neurons. This result can be verified and extended in a future study by direct examination of the temperature-entrained molecular phase in the various subsets of clock neurons.

In summary, our analyses revealed that thermocycle entrainment and photocycle entrainment produce very similar circadian expression profiles in fly heads, and that under common natural conditions light and temperature are expected to entrain both molecular and behavioral circadian rhythms cooperatively. As pointed out above, our results are in agreement with the notion that a single transcriptional clock is responsible for producing all light-entrained and temperature-entrained circadian rhythms. Nevertheless, we cannot formally exclude the existence of a specialized temperature-entrained oscillator that is coupled to the general transcriptional clock circuits. Such a theoretical temperature-entrained oscillator could have eluded detection in our analyses if it was located outside the head or in a small subset of the cells in the head or if it produced non-transcriptional circadian signals. Elucidation of the mechanisms of thermocycle entrainment will constitute an important next step in defining the temperature-entrained circadian oscillator(s).

## Materials and Methods

### Fly strains.

The wild-type strains used were *yellow white (y w), Canton S, and cinnabar brown (cn bw);* in addition, comparative analyses were performed for *y w; tim^01^* arrhythmic mutant flies. The flies were raised on standard yeast cornmeal medium.

### Time course collection.

For light experiments (described previously; see [27,28]), adult flies were entrained to 12 h of light followed by 12 h of darkness (LD, where ZT0 is lights-on and ZT12 is lights-off) at 25 °C for 5 d and subsequently released into constant darkness (DD, where circadian time [CT]0 is subjective lights-off). They were harvested onto dry ice every 4 h (unless specified otherwise) during the last day of entrainment and for 1–2 d of DD.

Temperature experiments were conducted entirely in the dark except for the initial seeding of parental bottles. Adults (parental) were placed in fresh media and allowed to mate and lay eggs at 25 °C for 5 d. The parents were cleared and the next generation was kept at 25 °C until early pupal stages. Next, they were transferred to a temperature cycle of 12 h of 18 °C followed by 12 h of 25 °C (CA, where CA0 is onset of 18 °C and CA12 is onset of 25 °C) until eclosion occurred. The newly eclosed flies were transferred to fresh media and allowed to entrain for an additional 4 d and then released into a constant temperature of 25 °C (AA). They were harvested onto dry ice every 4 h during the last day of entrainment and for one to two and a half days in AA (as specified: Day 1 = AA2–22, Day 2 = AA26–46, partial Day 3 = AA50–58). The frozen flies were vortexed and passed through a series of sieves in order to isolate the heads for RNA or protein extraction.

### Behavioral analysis.

Individual flies were monitored and their locomotor activity was analyzed with the *Drosophila* Activity Monitoring System IV (TriKinetics, http://www.trikinetics.com). For LD/DD experiments, the flies were reared under standard lighting conditions and monitored at 25 °C in LD and/or DD as specified. Temperature experiments were conducted entirely in the dark (unless otherwise specified). The flies were raised at 25 °C until the pupal stage and were then entrained to CA until eclosion. Individual flies were monitored and their locomotor activity analyzed as above both during entrainment (CA) and free-run (AA). Period lengths were calculated using ClockLab Software (ActiMetrics, http://www.actimetrics.com).

### Northern blot analysis.

Total RNA was extracted from approximately 100 μl of adult heads per time point using either RNA-STAT60 (Tel-Test, Incorporated, http://www.tel-test.com) or guanidinium thiocyanate followed by centrifugation in Cesium chloride solution. 15–30 μg of total RNA was denatured for 5 min at 65 °C and resolved on a 1% formaldehyde-agarose gel (20 mM MOPS [pH7], 5 mM NaOAc, 1 mM EDTA). The resolved RNA was transferred to Nytran membrane (Schleicher & Schuell, http://www.arraying.com) in 10x SSC overnight. Probe templates were radiolabeled as specified for the DECAprime II kit (Ambion, http://www.ambion.com). Hybridizations were carried out at 55 °C in UltraHyb solution (Ambion) supplemented with denatured fish sperm DNA (Roche, http://www.roche.com). Radioactive signals on the blots were visualized and quantitated with either a Storm or Typhoon Phosphorimager (Molecular Dynamics) and the results plotted in Microsoft Excel (http://www.microsoft.com). Fourier analyses were also performed on the northern data as indicated.

### Western blot analysis.

Total protein was extracted from about 35 μl of adult heads per time point in 75 μl Head Extraction Buffer (100 mM KCl, 20 mM Hepes [pH 7.5], 10% glycerol, 10 mM EDTA [pH 8], 0.1% Triton X-10, 50 mM NaF, 1 mM DTT) with 1x protease and phosphatase inhibitors (Roche) using a handheld homogenizer (Kontes, http://www.kontes.com). Samples were centrifuged at 14,000 rpm for 15 min at 4 °C. The supernatant was transferred to a new tube and centrifuged as above for an additional 10 min. 15–30 μg of total protein were resolved on 6% SDS polyacrylamide gels and transferred to nitrocellulose membrane (Schleicher & Schuell). Membranes were blocked for at least 1 h at room temperature with 5% nonfat dry milk in 1x TBST. Primary antibodies were diluted in blocking solution (1:10,000 for α-PER [rabbit], 1:2,000 for α-TIM [rat]) and incubated with the membranes at 4 °C overnight. The membranes were washed three times for 10 min each in 1xTBST and incubated with secondary antibodies (1:10,000) (Jackson ImmunoResearch, http://www.jacksonimmuno.com) for 1 h at room temperature. The membranes were washed as before and detection was carried out using ECL (Amersham Pharmacia Biotech, http://www.gehealthcare.com).

### Reverse transcriptase-PCR analysis.

RNA was extracted with RNA-STAT60 (Tel-Test, Incorporated) in the same manner as for northern blots. cDNA was generated using the ThermoScript RT-PCR System (Invitrogen, http://www.invitrogen.com) as described by the manufacturer with one exception: cDNA synthesis was carried out at 50 °C for 90 min with Oligo(dT)20. cDNA (3 μl)was used in subsequent PCR reactions with AccuPrime Pfx DNA Polymerase (Invitrogen).

### Microarray experiments.

Two sets of *y w* flies and one set of arrhythmic mutant *y w*; *tim01* flines were collected as described above for 1 d in CA and 1 d in AA. An additional set of *cn bw* flies was collected for the first 2 d in AA. RNA was extracted from adult heads with guanidinium thiocyanate followed by centrifugation in Cesium chloride solution. 50 μg of the RNA was further purified over RNeasy columns (Qiagen, http://www1.qiagen.com) according to instructions from the manufacturer. 25 μg of the purified RNA was used to generate biotin-labeled cRNA probe as described in the Affymetrix GeneChip manual. T7-d24 primers (MWG Biotech, http://www.mwg-biotech.com), Superscript Choice (Life Technologies, Invitrogen), and enzymes from New England Biolabs (http://www.neb.com) were used to synthesize cDNA. The ENZO Bioarray High Yield RNA transcript labeling kit was used for in vitro transcription reactions. Hybridization, washing, staining, and scanning of the target cRNA to the Affymetrix *Drosophila* Genome 1 arrays were carried out according to the Affymetrix GeneChip manual.

### Microarray data analysis.

The robust multi-array average (RMA) single algorithm [61] was used to prepare microarray data from each experiment. Using Fourier analysis, 24-h spectral power (F24) was calculated for appended time course experiments as in [27]. To estimate probability values and FDR, a permutated background model was used, in which time ordering of the real data was permuted 1,000 times to give a background distribution of F24. These were divided into a number of quantiles equal to the original number of probe sets and compared to F24 from the real data. Two types of probability values were calculated in association with F24: local *p*-values assigned to each probe set represent the odds of observing a F24 score of equal or higher strength after random permutation of the time order for the data series of that probe set, whereas global *p*-values represent the odds of observing an equal or higher F24 in the distribution calculated after random permutation of the time order for the data series of all of the probe sets. The local *p*-values are used in the selections performed for Figures 3, 4, and 5, whereas the global *p*-values are used to describe the 24-h periodic expression programs as represented in the graphs of Figures 2 and 6. For the analyses in Figures 2 and 6, only probe sets for which more than half of the values exceeded a 20th percentile cut-off were considered. In Figure 2B–2E, the number of selected rhythmic transcript profiles is plotted as a function of the threshold that is applied to the global *p*-value. The FDR in Figure 2A simply represents the ratio of the number of transcript profiles selected from the randomly permuted dataset over the number of transcript profiles selected from the real data. The LD data used in Figures 2 and 6 represent the *y w* (1) and *y w* (3) time course datasets described in references [27,28], whereas the DD data in Figure 2 represent these time courses plus 2 d of data from [29] formatted as described previously in [27]; the DD_1_ dataset in Figure 6 is identical to the DD data in Figure 2, whereas the DD_2_ dataset consists of the *cn bw* time course described in [28] plus 2 d of data from [62] and the average for the day of data from [63]. To select the 164 temperature-driven transcripts illustrated in Figure 3 and Table S1, the local *p*-value for the CA (2x wt + 1x *tim^01^*) data had to be <0.05, the average absolute daily range of RMA expression values had to be >0.3 for both the CA (2x wt) and the CA (2x wt + 1x *tim^01^*) data and the *p*-value found for a Kruskal-Wallis test of significant variation with daily time across the CA (2x wt + 1x *tim^01^*) data had to be <0.025. To select the 143 temperature-entrained circadian transcripts illustrated in Figure 4 and Table S1, the local *p*-value for the CA/AA (2x/4x wt) data had to be <0.001, the average absolute daily range of RMA expression values had to be >0.3, and the *p*-value found for a Kruskal-Wallis test of significant variation with daily time across the CA/AA (2x/4x wt) data had to be <0.01, and, to avoid light-driven effects, the local *p*-value for the AA (4x wt) data had to be <0.05. The set of core circadian transcripts in Figure 5 and Table S1 was defined by the overlap of the 143 temperature-entrained circadian transcripts and a set of 172 light-entrained circadian transcripts that was defined based on the following criteria: local *p*-value for LD/DD (8x/9x wt) had to be <0.001 [27], the average absolute daily range of RMA expression values had to be >0.3, and the *p*-value found for a Kruskal-Wallis test of significant variation with daily time across the LD/DD (8x/9x wt) data had to be <0.05, and, to avoid light-driven effects, the local *p*-value for the DD (9x wt) data had to be <0.05. Analysis results are made available at http://biorhythm.rockefeller.edu.

## Supporting Information

Figure S1 Northern Blot Analyses of Predicted Temperature-Specific Circadian Transcripts Several transcripts predicted to oscillate only in (A) temperature cycles and not light or (B) LD cycles and not temperature were tested on northern blots with samples from an LD/DD or CA/AA time course, respectively. The data were quantitated and subject to Fourier analysis (see Materials and Methods). The bars below the plots denote the entrainment scheme, with the white bar indicating light time points, black bars indicating dark time points, gray bars indicating free-run time points taken during subjective light, red bars indicating thermophase time points, blue bars indicating crypophase time points, and violet bars indicating free-run time points taken during subjective cryophase at constant 25 °C. The experiment in the bottom left panel used a standard stepwise 12-h 18 °C/12-h 25 °C thermocycle, while the experiments in the bottom middle and right panels were performed in the 24-hr thermocycle that gradually ramps from 18 °C (middle of the cryophase) to 25 °C (middle of the thermophase) and back to 18 °C; normal temperature-entrained circadian transcript rhythms were observed for *tim* under these conditions (unpublished data). The different colored lines in the *CG8451* plot represent two different transcripts. For additional details please see http://biorhythm.rockefeller.edu.
(54 KB PDF)

Figure S2 Average Locomotor Activity Behavior in CA and LD Locomotor activity behavior is “advanced” (relative to the onset of the respective *Zeitgeber*) in temperature entrainment as compared to light entrainment. The average locomotor activity counts from a group of flies in LD (A; *n* = 16) and CA 12:12 (B; *n* = 16), along with standard error bars, are plotted. Entrainment conditions are denoted by the bars below each plot, with the white bar indicating light time points, the black bar indicating dark time points, the red bar indicating 25 °C time points, and the blue bar indicating 18 °C time points.
(32 KB PDF)

Figure S3 Core Clock Genes Are Driven by Temperature in Arrhythmic *tim^01^* Flies (A) Northern blots showing expression of several of the core clock transcripts in *tim^01^* flies in 2 d of temperature entrainment and 1 d of free-run. An *rp49*-specific probe was used as a loading control.
(B) Quantitations from (A) in the light blue line. For comparison, the wild-type profiles (from Figure 7) are shown in the dark blue line. The bars below the northern images and plots denote the entrainment scheme, with red bars indicating 25 °C time points, blue bars indicating 18 °C time points, and violet bars indicating free-run time points taken at 25 °C.
(368 KB PDF)

Figure S4 *per* and *tim* Are Differentially Regulated during Temperature Entrainment The quantitations for *per* (blue) and *tim* (pink) in LD/DD (A; from unpublished data) and CA/AA (B and C; from Figure 8 and unpublished data) are shown. (A) *per* and *tim* are tightly coupled at all times in LD/DD.
(B) In CA, peak *per* expression occurs several hours before peak expression of the predominant *tim* transcript. This differential expression is absent in AA.
(C) *per* cycles in phase with a novel transcript of *tim* during temperature entrainment. The bars below the plots denote the entrainment scheme, with the white bar indicating light time points, black bars indicating dark time points, gray bars indicating free-run time points taken during subjective light, red bars indicating 25 °C time points, blue bars indicating 18 °C time points, and violet bars indicating free-run time points taken during subjective 18 °C. The error bars in (B) and (C) indicate the SEM of three independent time course experiments.
(32 KB PDF)

Figure S5 PER and TIM Expression in CA/AA (A) Western blots showing PER and TIM in temperature entrainment and subsequent free-run.
(B) Quantitations from (A). The bars below the western images and plots denote the entrainment scheme, with red bars indicating 25 °C time points, blue bars indicating 18 °C time points, and violet bars indicating free-run time points taken during subjective 18 °C. At least two independent profiles were obtained. For additional details please see http://biorhythm.rockefeller.edu.
(868 KB PDF)

Table S1 Temperature-Driven and Temperature-Entrained Circadian Transcripts Are Representative of Diverse Biological Functions Temperature-driven transcripts, temperature-entrained circadian transcripts, and dually regulated temperature-driven and temperature-entrained circadian transcripts (there is oscillating expression in a temperature cycle in *tim^01^* flies and rhythmic expression in AA in wild-type flies) are indicated in red, blue, and black typeface, respectively. Transcripts that overlap with a set of 172 predicted light-entrained circadian transcripts are indicated with a yellow background fill.
(49 KB PDF)

### Accession Numbers

The microarray data generated for this study have been deposited under accession number GSE6542 in the Gene Expression Omnibus data repository (http://www.ncbi.nlm.nih.gov/geo). FlyBase (http://www.flybase.net) accession numbers for the *Drosophila* genes discussed in this paper are *Clk* (FBgn0023076), *cry* (FBgn0025680), *Pdf* (FBgn0023178), *Pdp1* (FBgn0016694), *per* (FBgn0003068), *tim* (FBgn0014396), and *vri* (FBgn0016076).

## Abbreviations

AA: ambient/ambient
CA: cool/ambient
CLK: Clock
CRY: Cryptochrome
CYC: Cycle
DD: dark/dark
FDR: false discovery rate
*frq*: *frequency*
LD: light/dark
LN: lateral neuron
LN_V_: ventral lateral neuron
*Pdp1*: *PAR-domain protein 1*
*per*: *period*
RMA: robust multi-array average
*tim*: *timeless*
*vri*: *vrille*
*wc-1*: *white collar-1*
*wc-2*: *white collar-2*
ZT: *Zeitgeber* time
