# Supplementary material for: Integration of Light and Temperature in the Regulation of Circadian Gene Expression in Drosophila
Source: PLoS Genet. 2007 Apr 6;3(4):e54. doi: 10.1371/journal.pgen.0030054 (PMC1847695; doi:10.1371/journal.pgen.0030054)

Supplemental Figure S5

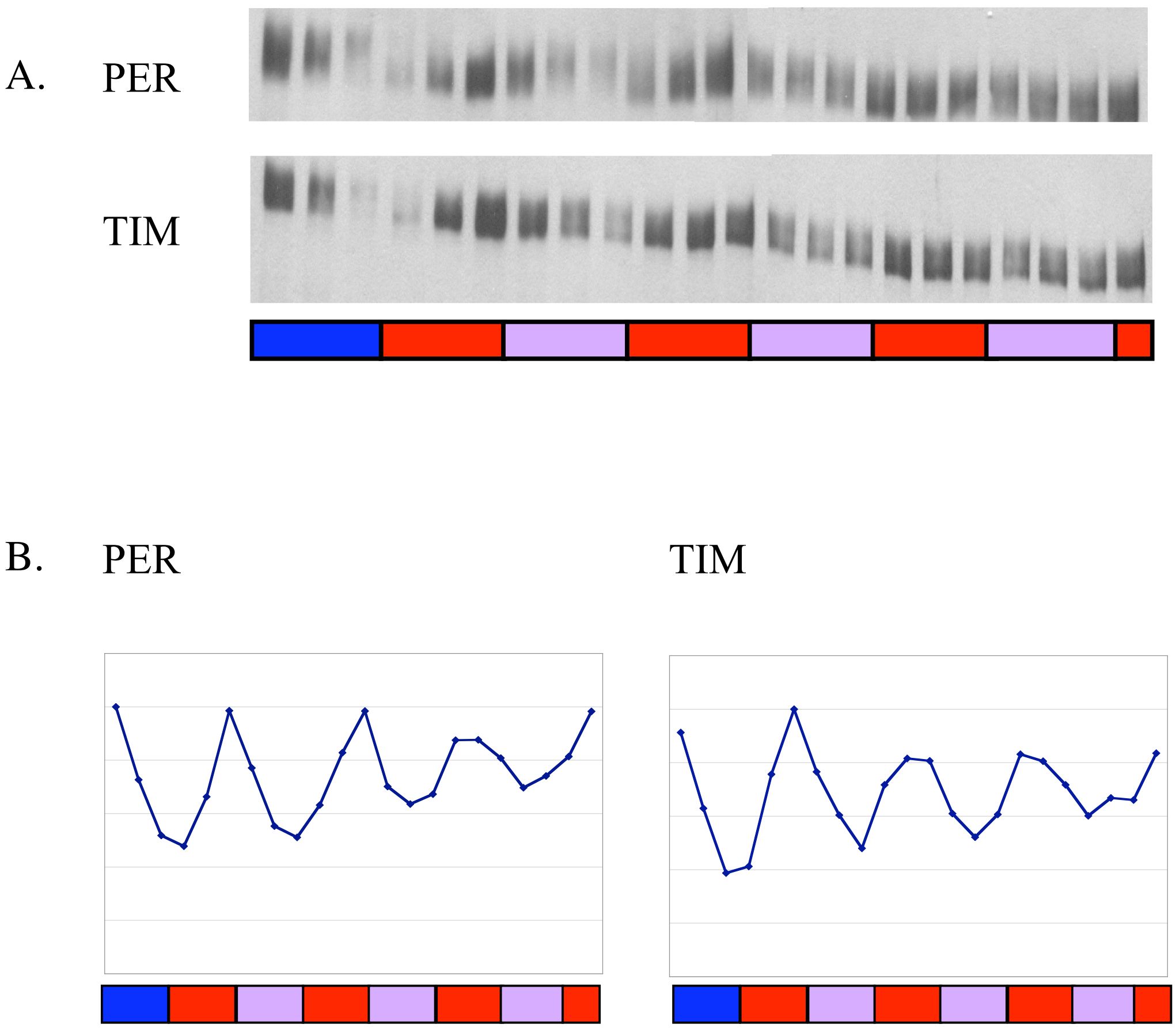

Supplement: Supplementary file 5 [file pgen.0030054.sg005.pdf]
