# Supplementary material for: Integration of Light and Temperature in the Regulation of Circadian Gene Expression in Drosophila
Source: PLoS Genet. 2007 Apr 6;3(4):e54. doi: 10.1371/journal.pgen.0030054 (PMC1847695; doi:10.1371/journal.pgen.0030054)
Supplement: Supplementary file 6 [file pgen.0030054.st001.pdf]

Supplemental Table S1

transport	development	nucleic acid metabolism	signal transduction	carbohydrate metabolism	folate metabolism
Ca-α1D	ash2	CG3800	activin-β	CG12030	CG12116
CG11642	Ca-α1D	Clp	CG14527	CG33138	Nmdmc
CG11739	CG12896	qkr58E-3	CHORD	CG4484	**other/unknown**
CG13848	CG32384	Tis11	drpr	CG5171	CG11275
CG33214	CG9650	CG2277	Fsh	CG6126	CG11395
CG4484	cpo	Ent2	fus	LCBP1	CG12269
CG4822	cpo	Reg-3	GstD10	sll	CG12868
CG4822	Crc	**(transcription)**	Hn	CG15096	CG13215
CG5885	drpr	ab	Hsp83	CG6218	CG13315
CG6126	E(bx)	Adf1	Ilp3	CG9886	CG13694
CG8051	fru	ash2	lgs	ATPCL	CG13845
CG8468	kn	CG1102	Rel	CG1213	CG13901
CG8498	lgs	CG9650	spz	CG15096	CG14084
CG8790	mbf1	Dsp1	wun2	CG3036	CG1416
Crc	oc	E(bx)	Tis11	CG8036	CG14253
Lsp1	px	Ell	Clk	Gasp	CG14693
mAcR-60C	rost	Ets65A	Hmgs	Nmdmc	CG15917
sll	SelD	HmgD	Csk	Sodh-1	CG17032
CG15096	Sox21b	HmgZ	Fsh	Tal	CG17230
Ndae1	SoxN	kn	inx2	**cytoskeleton**	CG17233
CG6600	sr	Max	Jheh2	form3	CG18600
CG10237	Sry-delta	mbf1	ogre	Hsp83	CG2081
CG17108	Vhl	Rel	smp-30	sll	CG31781
CG31085	wun2	Sox102F	TpnC25D	Vhl	CG3224
glob1	Hmgs	Sox21b	**(odorant binding)**	jbug	CG3226
CG10950	vri	SoxN	Or92a	Myo61F	CG4786
CG1213	B4	sr	Obp99a	Skeletor	CG6503
CG3036	CG13101	Sry-delta	Obp56h	spir	CG6985
CG3823	CG1942	Tudor-SN	**(synaptic transmission)**	**behavior**	CG7049
CG5404	Dr	Clk	mAcR-60C	Adf1	CG7607
CG8451	inx2	tim	cpo	Dat	CG8044
Ork1	Kr-h1	vri	cpo	Ddc	CG8187
**defense/immune response**	Myo61F	Kr-h1	fru	drpr	CG8408
CG12896	ogre	Dr	GRHR	fru	CG9511
CG6429	smp-30	**protein folding**	CG5618	Hsp83	CG9689
Cyp6g1	W	Cct5	Ork1	oc	CG9837
GstD10	**exoskeleton**	CG8258	**steroid metabolism**	ple	CR31541
Hop	CG4784	Crc	Cyp313a1	ogre	CG17124
Hsc70Cb	LCBP1	Hop	Cyp4e3	**(circadian rhythm)**	CG3348
Hsp83	Gasp	Hsc70Cb	Cyp6a8	Clk	CHkov1
MP1	**lipid/fatty acid metabolism**	Hsp83	mdy	tim	CG10175
psh	CG33110	DnaJ-1	mdy	vri	CG10553
Rel	CG3488	**proteolysis**	Ugt35a	**protein translation**	CG10560
spz	CG4822	As	CG7724	bru-3	CG11891
DnaJ-1	CG4822	CG1102	Cyp18a1	CG11642	CG13631
Ugt35a	CG5009	CG11459	Cyp6a21	CG32281	CG14275
CG3759	CG5508	CG11842	Hmgs	l(2)01424	CG1441
Ugt35b	CG8498	CG14523	Ugt35b	mdy	CG17325
CG10345	CG9009	CG14527	Cyp305a1	mdy	CG5156
CG17575	wun2	CG14528	**amino acid metabolism**	**takeout family**	CG7267
CG9027	CG33110	CG9377	CG32281	CG7079	CG10479
CG9649	CG3376	CG9649	CG6188	CG11852	CG11347
Eip71CD	Pdh	l(2)05070	Dat	CG14661	CG13360
GstE2	Cct2	oc	Ddc	CG5945	CG13510
Jheh2	CG11407	psh	Faa	to	CG31076
Mdr49	CG5853	ref(2)P	Hn	CG31207	CG32548
Ugt86Da	CG6409	UbcD4	SelD	CG5867	CG5237
Ugt86De	CG8093	Vhl	CG5793	**metabolism (other)**	CG5399
**protein modification**	CG9914	CG5618	Fmo-1	CG12162	CG6836
Nat1	v(2)k05816	CG5798	CG11796	CG14946	CG7047
EDTP	**muscle contraction**	Dip-B	CG15093	Cyp4d21	CG7510
CaBP1	Ca-α1D	CG16749	CG9363	CG3609	CG8343
CG8485	Scp2	CG4259	Fmo-2	CG3699	CG9119
Csk	CG4928	CG6465	hgo	Cyp12d1-p	CG9483
**1-carbon compound metabolism**	CG2121	CG9673	ple	Cyp12d1-p	CG9812
CG3940	CG13366		CPTI		
CG6074	TpnC25D		mnd		
